# Determinants of Full Vaccination Coverage among Children Aged 12-23 Months in Bangladesh: A Comparison between High- and Low-Performing Divisions

**DOI:** 10.1155/2024/7787593

**Published:** 2024-05-15

**Authors:** Moriam Khanam, Nahid Akhter Jahan

**Affiliations:** Institute of Health Economics, University of Dhaka, Dhaka 1000, Bangladesh

## Abstract

**Introduction:**

A better understanding of the significant factors behind childhood vaccination is important for designing strategies to increase vaccination coverage and reduce child mortality and morbidity. The study is aimed at identifying the determinants of full vaccination coverage among children aged 12-23 months in Bangladesh and at comparing the determinants between high- and low-performing areas.

**Methods:**

This study used the latest available Bangladesh Demographic and Health Survey 2017-18 data. A weighted sample of 1678 children was included in this study. The association between full vaccination coverage and explanatory variables was identified using chi-square test. Multivariable logistic regression analysis was employed to identify associated factors of full vaccination coverage.

**Results:**

Findings showed that about 88% of the children had full vaccination coverage. The odds of full vaccination coverage was significantly higher among children of mothers with secondary education compared to children of mothers with no formal education (AOR = 2.07, 95%CI = 1.16 to 3.70). Mother's working status was another significant factor behind full vaccination coverage (AOR = 1.53, 95%CI = 1.002 to 2.34). In addition, we identified that higher age of mother (AOR = 2.76, 95%CI = 1.28 to 5.96 for 20-34 years group and AOR = 12.14, 95%CI = 1.21 to 122.41 for 35 and above age group) and being in middle-income household (AOR = 4.66, 95%CI = 1.33 to 16.34) were significantly associated with full vaccination coverage in high-performing areas. On the other hand, children of mothers with secondary education level (AOR = 2.31, 95%CI = 1.86 to 4.49) and exposure to media (AOR = 1.58, 95%CI = 1.001 to 2.50) had higher odds of having full vaccination coverage in low-performing areas.

**Conclusions:**

This study identified the associated factors of full vaccination coverage among children. The findings indicate the importance of maternal education and mothers' employment for children's vaccination uptake. In low-performing areas, investment in education and awareness raising initiatives may play instrumental role in achieving full vaccination coverage.

## 1. Introduction

Immunization of children is considered as the safest method for protecting them from life-threatening diseases [1]. It has also been recognized as the most cost-effective and successful intervention in reducing childhood mortality and morbidity [2, 3]. The Expanded Program on Immunization (EPI) was formally established in 1974 with support from the World Health Organization to provide all basic vaccines and immunize children against key vaccine-preventable diseases around the globe [2, 4, 5]. Immunization currently prevents 2-3 million deaths per year; however, 1.5 million additional deaths could be avoided by expanding global vaccination coverage [6]. Although the rates of vaccine-preventable diseases have declined globally over the past few decades, many children remain unvaccinated especially in low- and middle-income countries [4]. Almost all zero-dose children live in low- and middle-income countries, particularly in Africa and Southeast Asia, with 11 million (62% of the total zero-dose children) living in only ten countries [7]. The number of zero-dose children was highest in India (2.7 million) followed by Nigeria (2.2 million), Indonesia (1.1 million), Ethiopia (1.1 million), and the Philippines (1.0 million) [8]. As of 2018, an estimated 700,000 under-five children died from vaccine-preventable diseases, and most of them are from low- and middle-income countries [9]. Therefore, to achieve the health-related Sustainable Development Goals (SDGs), especially the target of reducing preventable deaths of newborns and under-five children by 2030, it is crucial to expand the coverage of vaccination of children. More than 50 million deaths can be avoided through childhood vaccination, and measles vaccine can prevent 19 million deaths between 2023 and 2030 [7].

The percentage of children aged 12-23 months in the world receiving measles vaccine increased gradually from 73% in 1990 to 84% in 2010 and 86% in 2019 [10]. However, the percentage of children receiving measles vaccine reduced during and after the COVID-19 pandemic and was 82% in 2021. The trend of coverage of measles vaccine was very similar in low- and middle-income countries, and the figures were marginally lower than the global average. The percentage of children aged 12-23 months receiving measles vaccine was 81% in 2021 in low- and middle-income countries [10].

Compared to other South Asian countries, the vaccination coverage for children is the highest in Bangladesh [11]. Childhood vaccination prevents roughly 200,000 deaths in Bangladesh each year [12]. Despite the noteworthy success, Bangladesh is on the list of the top ten countries with the highest childhood mortality globally [4, 13]. The percentage of children aged 12-23 months receiving measles vaccine was 89% in 2021, and the full vaccination coverage of children aged 12-23 months remained stagnant for quite a few years in Bangladesh [14]. The percentage of children aged 12-23 months receiving all basic vaccinations was 86% in 2011, which reduced to 83.8% in 2014 and again increased to 89.1% in 2017-18 [15–17]. The country is facing hindrances in reducing dropouts, invalid doses, and geographical inequity in full vaccination coverage despite all the supply-side interventions with free vaccination services. Dropout from Penta1 to Penta3 reduced from 2.0% in 2010 to 1.3% in 2019 and Penta1 to MR1 from 6.0% in 2010 to 4.6% in 2019. Invalid doses were found to be the most prominent one for MR1 (7.8%), and 3.7% of the children received invalid MR2 [18]. Full vaccination coverage was highest in the Rajshahi Division (93.1%) and lowest in the Sylhet Division (85.9%) [15]. Therefore, it is important to look again into the demand-side factors using the latest available dataset to identify area-wise key factors determining the full vaccination coverage. It is important to design strategies and policies to ensure completion of all recommended vaccines of children to further reduce childhood morbidity and mortality [13]. Bangladesh, with 171.2 million people in 2022, was in the eighth position among the countries with largest population in the world [19]. The total number of under-five children was 16.3 million [20]. Hence, even a small percentage of unvaccinated children in Bangladesh implies that many children are at risks of vaccine-preventable diseases. Moreover, the vision of the 5.0 strategy of Gavi, the Vaccine Alliance, is to leave no one behind with immunization by 2030 [21]. A better understanding of the influencing factors of childhood vaccination is crucial for designing proper strategies to improve equity in immunization coverage and to reach all zero-dose children.

Evidence shows that there are various socioeconomic and demographic determinants of health along with the healthcare seeking behavior of the households. This reinforced the importance of a multisectoral approach in designing policies to achieve health-related sustainable development goals and universal immunization coverage in low- and middle-income countries. Several studies have been conducted in Bangladesh and other South Asian countries which identified sex of children [22], mother's age [5, 11], mother's education [2, 4], mother's employment status [5], mother's number of antenatal care visits [23], whether the delivery of child was at health facility [5, 11, 23], wealth status of household [2, 13, 24], household's size [2, 5], and region of residence [4] as the influential factors behind childhood immunization. Although there are various studies in Bangladesh and other similar contexts vary, few studies focused on full vaccination of children from the age group 12-23 months. In addition, most of the studies in Bangladesh were done using data from the Bangladesh Demographic and Health Survey (BDHS) 2014 or before. Moreover, some studies were conducted in specific geographic settings rather. To the best of our knowledge, no studies have been conducted for identifying the factors influencing the full vaccination coverage in low-performing and high-performing divisions in Bangladesh. Therefore, the objective of this study is to identify the full vaccination coverage and its associated factors among children aged 12-23 months in Bangladesh, using the latest available BDHS 2017-18 dataset. In addition, this study identified the determinants across low- and high-performing divisions. This would provide the updated vaccination coverage situation, the existing geographical inequity, and its associated factors in Bangladesh. This dataset is the nationally representative dataset, and the findings are generalizable for the children aged 12-23 months in Bangladesh. The study attempted to identify the determinants of full vaccination coverage in both low- and high-performing divisions to aid in designing relevant strategies and policies for achieving universal vaccination coverage in the country.

## 2. Materials and Methods

### 2.1. Data Source, Study Design, and Study Participants

This study used data from the BDHS 2017-18 to identify the associated factors of childhood vaccinations in Bangladesh. The DHS program of the USA conducted this study, and the National Institute of Population Research and Training (NIPORT) of the Ministry of Health and Family Welfare (MoHFW) of Bangladesh was the local partner. A two-stage stratified random sampling procedure was followed for data collection. In the first stage, 672 enumeration areas (EAs) were selected using probability proportional. At the next stage, 30 households were selected on average from each EAs using systematic selection method. The survey interviewed a total of 20,127 reproductive-aged (15-49 years) ever-married women with a response rate of 99%. BDHS 2017-18 is a nationally representative cross-sectional survey which collects information on various indicators including demographic and socioeconomic characteristics, maternal and child health, immunization of children, child feeding practices, and biomarkers. Information on vaccination coverage was collected in two ways: either from vaccination cards or from mother's verbal reports. The details on sampling procedure, data collection instruments, and data collection methods are described elsewhere [15]. This dataset is publicly available at the DHS website (https://dhsprogram.com/data) for researchers. We sought permission and obtained this dataset from the DHS authority.

This study included data of children in the age group 12-23 months. Therefore, children from other age groups were excluded. In addition, children with incomplete data on the outcome and explanatory variables were excluded from this study.

### 2.2. Ethical Approval and Informed Consent

BDHS followed standardized procedure for data collection, and informed consent was taken from each respondent during data collection. Hence, no additional ethical review was required for this study. However, we sought approval from the DHS authority for using and analyzing the dataset for this study.

### 2.3. Outcome Variable

The outcome variable of this study is full vaccination coverage of children aged 12-23 months. As used in other studies, we defined full vaccination coverage as whether the child received at least one dose of BCG vaccine, three doses of DPT-containing vaccine, three doses of polio vaccine, and one dose of measles-containing vaccine or not [25, 26]. We coded this variable as “1” if the child received full vaccination and “0” otherwise.

### 2.4. Explanatory Variables

Various studies identified that vaccination of children is affected by both individual and community level factors [2, 5, 27]. The individual level variables in this study included sex of child (male or female), age of mother (15-19 years, 20-34 years, and 35 and above), education of mother (no education, primary, secondary, and tertiary), mother's working status (whether mother was working or not), mother's exposure to media (“yes” if read newspaper and/or listen to music and/or watch TV at least once a week and “0” otherwise), number of antenatal care (ANC) visits (categorized as none, 1-3, and 4 and above), place of delivery at birth (at home or at health facility), household size (grouped as small if 1-4, medium if 5-6, and large if above 6), and wealth status of household (grouped into five quintiles: poorest, poor, middle, richer, and richest based on household's durable and nondurable asset holdings). Region of residence (rural or urban) and administrative division (defined as eight administrative divisions of Bangladesh) were the two community level variables. The selection of explanatory variables and their categorization were done based on the relevant existing literature and the availability of BDHS 2017-18 datasets [2–5, 11, 13, 15, 24, 25, 27, 28].

### 2.5. Statistical Analysis

Descriptive analyses such as cross tabulations were conducted to report frequencies and percentages of vaccination coverage across different sociodemographic and economic characteristics of children aged 12-23 months. Association between each explanatory variable and vaccination coverage was determined using the chi-square test in the bivariate analysis. As the dependent variable is binary, we used binary logistic regression analysis to find out the associated factors of vaccination coverage among children. The logistic regression analysis uses the maximum likelihood method for estimating the parameters [29]. We used two multivariable logistic regression models in this study. The first one included explanatory variables which were significant at *p* < 0.2 level in the bivariate analysis [30]. The second one included all the available explanatory variables irrespective of their significance in the bivariate analysis. In addition, we tried to identify whether the factors of full immunization coverage vary across the low-performing and high-performing divisions. Therefore, we divided the sample into two groups using 90% full vaccination coverage as the benchmark. In one group, we included children from divisions with 90% and above vaccination coverage, and the other group included children from divisions with less than 90% coverage. In the multivariable models, statistical significance was determined with *p* values < 0.05. We reported results with adjusted odds ratios (AORs) and 95% confidence intervals. We also conducted Wald tests to identify the overall significance of the categorical variables. This study used Hosmer–Lemeshow test to assess the goodness of fit of our models as suggested in literature [31]. This study used proper sampling weight as suggested by DHS to make the sample more representative of the population at the national level. The “svy” command was used to assign the weight of the sample to reduce clustering and sample stratification. We analyzed data for this study using STATA 16.0 software.

## 3. Results

### 3.1. Vaccination Coverage among Children according to Background Characteristics

The weighted sample size of this study was 1678 children.  Table 1 represents the vaccination coverage across different demographic and socioeconomic characteristics. The study found that about 88% of the children received all basic vaccination. The distribution of the vaccination coverage was similar across genders and geographical regions. The percentage of vaccination coverage of children increases with the increase in mothers' age. According to mother's education, the percentage of children aged 12-23 months who received all basic vaccination coverage was the highest among children of mothers with higher education level (91.06%), while this rate was the lowest among children of mothers with no formal education (80.61%). The percentage of vaccinated children was significantly higher among those children whose mothers had access to print or electronic media at least once a week compared to those whose mothers did not have such access (90.13% versus 85.85%, *p* = 0.02). The likelihood of vaccination coverage significantly increased with their mothers' number of ANC visits as the coverage was about 91% among children whose mothers had 4 or more ANC visits, followed by about 86% and 84% among children of mothers with 1-3 ANC visits and no ANC visit, respectively. Similarly, the proportion of vaccinated children was significantly higher among children who were born with institutional delivery compared to children who born at home (90.62% versus 85.85%, *p* = 0.01). Vaccination coverage of children was about 90% among children of mothers who were working, while this coverage was about 87% among children whose mothers were not working. The proportions of vaccinated children were similar across household sizes. The vaccination coverage was the highest (91.26%) among children who belonged to the richest households, while the coverage was the lowest (85.82%) among children who belonged to poorer households. The highest vaccination coverage was in Rajshahi Division (91.6%), and the lowest coverage was in Sylhet Division (84.3%).

### 3.2. Determinants of Full Vaccination Coverage among Children

After including those variables in the multivariable logistic regression model which were found significant at 20% level in the bivariate analysis, this study found the mother's level of educational attainment and working status as significantly associated with childhood vaccination in Bangladesh (Table 2). Compared to children of mothers with no formal education, children of mothers with a secondary level of education had 2.07 times greater odds of having all basic vaccination coverage, and the result was statistically significant (AOR = 2.07, 95%CI = 1.16 to 3.70, *p* = 0.014). The Wald test for the joint significance of mother's education shows that there is significant association between all basic vaccination coverage and mother's level of education (*p* = 0.021). Children of mothers who were found employed at the time of data collection had significantly 53% higher odds of being vaccinated compared to children whose mothers were not employed (AOR = 1.53, 95%CI = 1.002 to 2.34, *p* = 0.049).

When we included all selected variables irrespective of their significance from bivariate analysis, we found mothers' level of educational attainment as significantly associated with full vaccination coverage of children aged 12-23 months (*p* value from the Wald test = 0.015) (Table 3). Children of mothers with higher educational attainment had about 93% higher odds of being vaccinated compared to those whose mothers had no formal education; however, the result was weakly significant at 10% level (AOR = 1.93, 95%CI = 0.90 to 4.13, *p* = 0.09). The odds of being vaccinated was 2.26 times greater among children whose mothers received secondary level of education compared to children with mothers having no formal education, and the result was highly significant at 1% level (AOR = 2.26, 95%CI = 1.23 to 4.14, *p* = 0.008).

From the high-performing divisions, this study found that mother's age (*p* value from the Wald test = 0.013) is significantly associated with full immunization coverage (Table 4). Children of mothers from the 20-34 years age group had above 2 times greater odds of receiving full vaccination (*p* < 0.01) compared to children of mothers from the 15-19 years age group. Similarly, children whose mothers were in the 35 and above years age group had about 12 times higher odds of receiving full vaccination (*p* = 0.034) compared to those with mothers in the 15-19 years age group. In addition, children who belonged to the middle-income households had significantly higher odds of receiving vaccines compared to those from households in the poorest wealth quintiles (AOR = 4.66, 95%CI = 1.33 to 16.33, *p* = 0.016). However, from the Wald test, we did not find any evidence of the association between the wealth status of household and vaccination coverage (*p* = 0.119).

From the low-performing divisions, we found that mother's education level and their exposure to media were significantly associated with full vaccination coverage. Children whose mothers had a secondary level of educational attainment had significantly greater odds of being fully vaccinated compared to those whose mothers had no education (AOR = 2.31, 95%CI = 1.19, 4.49, *p* = 0.014). Children of higher educated mothers had above 2 times greater odds of being fully vaccinated compared to children of mothers with no education; however, the result was weakly significant (AOR = 2.21, 95%CI = 0.92 to 5.27, *p* = 0.075). The Wald test shows that overall mother's education level is significantly associated with all basic vaccination coverage of children (*p* = 0.048). This study also found that children of mothers who had exposure to media had 1.5 times higher odds of having full vaccination coverage compared to children whose mothers did not have media exposure (AOR = 1.58, 95%CI = 1.001 to 2.50, *p* = 0.047).

From the Hosmer–Lemeshow test, we found no evidence of poor fit of our logistic regression models.

## 4. Discussion

This study used the BDHS 2017-18 dataset and used multiple logistic regression analysis to identify the associated factors of childhood immunization in Bangladesh. This study also identified the associated factors of vaccination coverage from low-performing divisions and high-performing divisions. The study found that about 88% of the coverage of full vaccination was among children aged 12-23 months in Bangladesh. The findings from other studies and survey reports show that the coverage of full vaccination of children has been almost constant for several years and it has been less than 90% [2, 26]. The current study also found that the coverage of full vaccination among this group of children was slightly over 90% in Rajshahi, Khulna, and Rangpur divisions. On the other hand, Dhaka Division had 88.7% vaccination coverage for children followed by 86.61% in Chittagong division, 86.29% in Mymensingh division, 86.14% in Barisal Division, and 84.3% in Sylhet Division. The country needs to design policies to reach all the children to be aligned with the Immunization Agenda 2030 and the vision of the 5.0 strategy of Gavi, the Vaccine Alliance [21]. Identifying the factors influencing childhood immunization would be helpful to adopt area-specific appropriate strategies and interventions to improve immunization coverage in Bangladesh.

The study identified mother's educational attainment and working status as significantly associated with all basic vaccination coverage among children aged 12-23 months in Bangladesh. We found that compared to children of mothers with no education, the odds of receiving all basic vaccination was higher among children of mothers with a secondary level of education. This study finding is consistent with findings from other studies in different settings including Bangladesh. Banerjee et al. [24] found that the likelihood of being vaccinated increases with greater maternal education compared to no education in Bangladesh and India. Another study in Bangladesh also found similar results [2]. Moreover, a study in Ethiopia found secondary maternal education as a significant factor behind full childhood immunization [27]. The reasoning behind this might be that educated mothers' have better knowledge about the benefits of vaccination of children and its schedules than noneducated parents [2, 24, 27]. Bangladesh has made remarkable progress in improving women's education over the past several years [32]. Our study findings imply that further improvement in maternal education could improve the immunization coverage of children. Bangladesh should reinforce the existing female secondary stipend program to assist in increasing the female students' enrolment and retention in secondary schools. Furthermore, more awareness raising initiatives, especially targeting mothers on vaccination of children, could help improving the coverage. Strengthening community-based health system could play crucial role in reaching mothers and children for increasing the vaccination coverage.

The current study also identified that children of mothers who were working for income generation had greater odds of receiving all basic vaccination compared to children of mothers who were not working. A study in Bangladesh also found a positive association between mother's working status and childhood vaccination [24]. Another study in Bangladesh identified that children of unemployed mothers had greater odds of failing to receive BCG and measles vaccines [5]. However, another study in Bangladesh did not find any significant association between mother's working status and vaccination of children [4]. There is also evidence from urban slums of Bangladesh that children whose mothers work outside home had a lower likelihood of receiving BCG vaccine [3]. The possible explanation of our study finding could be that employed mothers have greater autonomy in taking their children to vaccination centers. There is evidence that in children whose mothers have autonomy in decision making, their likelihood of receiving vaccines increased [2, 13]. Moreover, working mothers might have better exposure to vaccine related information at their workplaces. This finding implies the need of increasing female labor force participation in Bangladesh.

This study also found that children of mothers with higher age group and belonging in middle-income households had higher chances of full immunization coverage in the high-performing divisions. Therefore, policies targeted to young mothers and low-income families might improve the vaccination coverage further in high-performing areas to reach a universal vaccination coverage. The higher aged mothers might have better knowledge on vaccination of children. Our study findings also showed that children of mothers with higher education level and exposure to media had higher odds of having full vaccination coverage in low-performing divisions. This implies that lack of knowledge of mothers about the importance of vaccinating their children might be a reason behind lower vaccination coverage. This finding has important policy implications. Planned investment in female education and awareness creation activities, especially in low-performing divisions, is expected to reduce the geographical inequity and increase full vaccination coverage in Bangladesh.

This study has several limitations. Firstly, as the data was cross sectional in nature, causal relationship between vaccination coverage of children and the explanatory variables may not be explored. Secondly, the vaccination status of children was measured based on either vaccination cards or from mothers' self-reporting. Hence, recall bias and its potential effects cannot be ignored. Moreover, due to data limitation, this study could not capture supply-side factors of childhood immunization. Despite these limitations, this study has some key strengths. Firstly, it used a large, nationally representative data. Therefore, the study findings could be generalized for the children aged 12-23 months in Bangladesh. Secondly, this study considered a large number of variables in the multivariable logistic regression analysis, which strengthens the validity of the study findings.

## 5. Conclusions

Our study findings indicate that the full vaccination coverage among children aged 12-23 months in Bangladesh is high. However, some groups need special attention to achieve health-related Sustainable Development Goals and leave no one behind. The educational attainment of mothers and their working status significantly influence the vaccination coverage of the children. Further, the mother's age and belonging to middle-income household were significant factors in high-performing divisions, while the mother's education and exposure to media were significant factors for low-performing divisions. Increasing the vaccination coverage in the low-performing areas shall reduce inequity in vaccination in Bangladesh and raise the country's average vaccination coverage closure to 95% of the national target. Therefore, proper policies should be designed targeting these groups to reach full vaccination coverage among children in Bangladesh. Future research could further investigate the factors influencing the dropouts and invalid doses in vaccination in Bangladesh, which are crucial for attaining full immunization coverage and avoiding vaccine-preventable deaths, using quantitative and qualitative data.

## Figures and Tables

**Table 1:** Background characteristics of the study participants and their vaccination status.

Variables	All basic vaccinations (%)	Total number of children (*n*)
*Sex*		
Male	87.51	841
Female	89.02	837
*p* value	0.38	
*Age of mother*		
15-19 years	86.98	297
20-34	88.31	1269
35 and above	91.08	112
*p* value	0.58	
*Education of mother*		
No education	80.61	102
Primary	84	477
Secondary	90.74	795
Higher	91.06	304
*p* value	<0.001	
*Having media exposure*		
Yes	90.13	946
No	85.85	732
*p* value	0.0245	
*No. of ANC visits*		
None	83.67	125
1-3	85.76	751
4 and above	91.32	802
*p* value	0.0028	
*Place of delivery*		
At home	85.85	840
At health facility	90.62	838
*p* value	0.0143	
*Mother's working status*		
Yes	90.02	628
No	87.21	1050
*p* value	0.1718	
*HH size*		
Small (up to 4)	88.04	530
Medium (5-6)	88.13	605
Large (above 6)	88.63	544
*p* value	0.9574	
*Wealth status*		
Poorest	86.63	340
Poorer	85.82	348
Middle	90.39	321
Richer	87.1	336
Richest	91.26	333
*p* value	0.16	
*Division*		
Barisal	86.14	94
Chittagong	86.61	354
Dhaka	88.7	425
Khulna	91.31	144
Mymensingh	86.29	141
Rajshahi	91.68	188
Rangpur	90.17	190
Sylhet	84.3	143
*p* value	0.51	
*Region of residence*		
Urban	88.26	450
Rural	88.26	1228
*p* value	0.99	
*Total*	88.26	1678

**Table 2:** Multivariable analysis of factors associated with childhood vaccinations (including explanatory variables which were significant in bivariate analysis).

Variables	Odds ratio	*p* value	95% confidence interval	
			Lower limit	Upper limit
*Mother's education*				
No education (ref)				
Primary	1.200	0.520	0.688	2.096
Secondary	2.071	0.014	1.158	3.703
Higher	1.794	0.130	0.841	3.827
		0.021^∗^		
*Working status of mother*				
Not working (ref)				
Working	1.531	0.049	1.003	2.339
*Wealth status*				
Poorest (ref)				
Poorer	0.788	0.360	0.473	1.313
Middle	1.068	0.809	0.626	1.822
Richer	0.711	0.263	0.391	1.293
Richest	0.968	0.928	0.475	1.973
		0.541^∗^		
*Place of delivery*				
At home (ref)				
At health facility	1.307	0.229	0.845	2.022
*Number of ANC visits*				
No ANC visit (ref)				
1-3	1.032	0.917	0.574	1.853
4 and above	1.475	0.240	0.770	2.826
		0.146^∗^		
*Mother's exposure to mass media*				
No (ref)				
Yes	1.217	0.350	0.805	1.839

^∗^ *p* value from the Wald test.

**Table 3:** Multivariable analysis of factors associated with childhood vaccinations (including all explanatory variables).

Variables	Odds ratio	*p* value	95% confidence interval	
			Lower limit	Upper limit
*Sex of child*				
Female (ref)				
Male	0.859	0.382	0.610	1.208
*Age of mother*				
15-19 (ref)				
20-34	1.179	0.473	0.751	1.854
35 and above	1.899	0.130	0.827	4.364
		0.314^∗^		
*Mother's exposure to mass media*				
No (ref)				
Yes	1.175	0.445	0.777	1.777
*No of ANC visits*				
None (ref)				
1-3	0.971	0.923	0.531	1.773
4 and above	1.397	0.323	0.719	2.717
		0.151^∗^		
*HH size*				
Small (up to 4)	1.037	0.860	0.689	1.561
Medium (5-6)	1.054	0.825	0.658	1.689
Large (7 and above) (ref)				
		0.972^∗^		
*Place of delivery*				
At home (ref)				
At health facility	1.287	0.261	0.828	1.999
*HH wealth status*				
Poorest (ref)				
Poorer	0.746	0.262	0.446	1.246
Middle	1.028	0.919	0.603	1.753
Richer	0.702	0.281	0.368	1.337
Richest	1.004	0.993	0.445	2.263
		0.476^∗^		
*Education level of mother*				
No education (ref)				
Primary	1.272	0.407	0.719	2.249
Secondary	2.261	0.008	1.234	4.141
Higher	1.932	0.090	0.902	4.136
		0.015^∗^		
*Working status of mother*				
Not working				
Working	1.446	0.114	0.914	2.288
*Division*				
Barishal (ref)				
Chittagong	0.976	0.947	0.477	1.998
Dhaka	1.286	0.495	0.623	2.654
Khulna	1.419	0.399	0.628	3.208
Mymensingh	1.055	0.884	0.516	2.157
Rajshahi	1.576	0.229	0.751	3.312
Rangpur	1.181	0.672	0.546	2.558
Sylhet	0.993	0.986	0.501	1.973
		0.91^∗^		
*Region of residence*				
Rural (ref)				
Urban	0.869	0.545	0.553	1.368

^∗^ *p* value from the Wald test.

**Table 4:** Factors associated with childhood vaccinations in high-performing and low-performing areas.

Variables	Divisions with 90% and above vaccination coverage (*n* = 522)			Divisions with less than 90% vaccination coverage (*n* = 1556)		
	Odds ratio	*p* value	95% CI [lower limit, upper limit]	Odds ratio	*p* value	95% CI [lower limit, upper limit]
*Sex of child*						
Female (ref)						
Male	0.798	0.508	[0.408, 1.561]	0.846	0.413	[0.565, 1.265]
*Age of mother*						
15-19 (ref)						
20-34	2.763	0.010	[1.281, 5.961]	0.699	0.238	[0.387, 1.267]
35 and above	12.144	0.034	[1.205, 122.414]	0.957	0.927	[0.37, 2.471]
		0.013^∗^			0.354^∗^	
*HH size*						
Small (up to 4) (ref)						
Medium (5-6)	0.999	1.000	[0.482, 2.073]	1.063	0.810	[0.644, 1.754]
Large (7 and above)	0.726	0.495	[0.288, 1.829]	1.193	0.489	[0.723, 1.969]
		0.758^∗^			0.779^∗^	
*Education level of mother*						
No education (ref)						
Primary	0.898	0.922	[0.102, 7.911]	1.356	0.332	[0.732, 2.510]
Secondary	1.976	0.544	[0.217, 17.977]	2.308	0.014	[1.186, 4.492]
Higher	1.019	0.988	[0.099, 10.525]	2.206	0.075	[0.923, 5.273]
		0.202^∗^			0.048^∗^	
*Working status of mother*						
Not working						
Working	1.593	0.267	[0.698, 3.638]	1.410	0.208	[0.825, 2.411]
*Mother's exposure to mass media*						
No (ref)						
Yes	0.539	0.162	[0.226, 1.284]	1.579	0.047	[1.001, 2.50]
*No of ANC visits*						
None (ref)						
1-3	1.889	0.366	[0.474, 7.535]	0.864	0.667	[0.442, 1.688]
4 and above	2.801	0.193	[0.591, 13.281]	1.239	0.568	[0.592, 2.594]
		0.386^∗^			0.273^∗^	
*Place of delivery*						
At home (ref)						
At health facility	0.899	0.814	[0.372, 2.179]	1.401	0.216	[0.821, 2.393]
*HH wealth status*						
Poorest (ref)						
Poorer	0.999	1.000	[0.382, 2.616]	0.759	0.365	[0.418, 1.380]
Middle	4.664	0.016	[1.332, 16.339]	0.668	0.21	[0.355, 1.257]
Richer	1.611	0.454	[0.459, 5.651]	0.589	0.161	[0.281, 1.236]
Richest	2.636	0.250	[0.503, 13.805]	0.736	0.502	[0.300, 1.805]
		0.119^∗^			0.633^∗^	
*Region of residence*						
Rural (ref)						
Urban	0.924	0.870	[0.359, 2.382]	0.957	0.868	[0.571, 1.603]

^∗^ *p* value from the Wald test.

## Data Availability

This dataset is publicly available in the DHS website (https://dhsprogram.com/data/).
